# Factors associated with syphilis incidence in the HIV-infected in the era of highly active antiretrovirals

**DOI:** 10.1097/MD.0000000000005849

**Published:** 2017-01-13

**Authors:** Mohaned Shilaih, Alex Marzel, Dominique L. Braun, Alexandra U. Scherrer, Helen Kovari, Jim Young, Alexandra Calmy, Katharine Darling, Manuel Battegay, Matthias Hoffmann, Enos Bernasconi, Maria C. Thurnheer, Huldrych F. Günthard, Roger D. Kouyos, and the Swiss HIV Cohort Study

**Affiliations:** a Division of Infectious Diseases and Hospital Epidemiology, University Hospital Zurich; b Institute of Medical Virology, University of Zurich, Zurich; c Basel Institute for Clinical Epidemiology and Biostatistics, University Hospital Basel, Basel; d Infectious Diseases Department, Genève University Hospital, Genève; e Infectious Diseases Department, Lausanne University Hospital, Lausanne; f Division of Infectious Diseases and Hospital Epidemiology, University Hospital Basel, Basel; g Division of Infectious Diseases and Hospital Epidemiology, Kantonal Hospital St. Gallen, St. Gallen; h Division of Infectious Diseases, Regional Hospital Lugano, Lugano; i Department of Infectious Diseases, Inselspital, Bern University Hospital, University of Bern, Bern, Switzerland.

**Keywords:** HIV, HIV coinfections, nevirapine, syphilis, syphilis prevention

## Abstract

After several years of steady decline, syphilis is reemerging globally as a public health hazard, especially among people living with human immunodeficiency virus (HIV). Syphilis resurgence is observed mainly in men who have sex with men (MSM), yet other transmission groups are affected too. In this manuscript, we study the factors associated with syphilis incidence in the Swiss HIV cohort study in the era of highly effective antiretrovirals. Using parametric interval censored models with fixed and time-varying covariates, we studied the immunological, behavioral, and treatment-related elements associated with syphilis incidence in 3 transmission groups: MSM, heterosexuals, and intravenous drug users. Syphilis incidence has been increasing annually since 2005, with up to 74 incident cases per 1000 person-years in 2013, with MSM being the population with the highest burden (92% of cases). While antiretroviral treatment (ART) in general did not affect syphilis incidence, nevirapine (NVP) was associated with a lower hazard of syphilis incidence (multivariable hazard ratio 0.5, 95% confidence interval 0.2–1.0). We observed that condomless sex and younger age were associated with higher syphilis incidence. Moreover, time-updated CD4, nadir CD4, and CD8 cell counts were not associated with syphilis incidence. Finally, testing frequency higher than the recommended once a year routine testing was associated with a 2-fold higher risk of acquiring syphilis. Condomless sex is the main driver of syphilis resurgence in the Swiss HIV Cohort study; ART and immune reconstitution provide no protection against syphilis. This entails targeted interventions and frequent screening of high-risk populations. There is no known effect of NVP on syphilis; therefore, further clinical, epidemiological, and microbiological investigation is necessary to validate our observation.

## Abstract 2

Supplemental Digital Content is available in the text

**Key Points**

- Syphilis coinfection in the HIV infected is rising due to an increase in condomless sex in the younger MSM population in Switzerland.
- Antiretrovirals provide no additional protection against syphilis except for provisional effect of Nevirapine.
- A protective effect of Nevirapine on syphilis has not been reported in the literature and could be due to confounding thus requires further investigation.

## 1 Introduction

Syphilis is a reemerging public health hazard that has been on the rise in recent years globally^[1]^ and locally in Switzerland.^[2]^ This is particularly the case for the human immunodeficiency virus (HIV)-infected population, with 1 review placing the mean prevalence of syphilis in the HIV-infected population at 9.5%.^[3]^ The importance of syphilis as a coinfection in HIV-infected individuals does not only stem from the negative effect of syphilis on the natural course of HIV infection (manifested as a temporary reduction in CD4 cells and an elevation in HIV viral load^[4,5]^) but also from the enhancement of HIV transmission in individuals coinfected with syphilis.^[6]^ It is estimated that 60% of syphilis cases are asymptomatic,^[2]^ and syphilis infection enhances HIV transmission and other coinfections (e.g., Hepatitis B^[7]^ and Hepatitis C^[8]^), which places syphilis coinfection in the forefront of HIV transmission and public health concerns.

Antiretroviral treatment (ART) has been shown to affect HIV and some of its coinfections through 3 main axes: immunological, behavioral, and direct. On the immunological front, ART enhances immune reconstitution in HIV-infected individuals leading to an enhanced protection against pathogens.^[9]^ More generally, there is a strong interaction between the immune system and syphilis (e.g., a low CD4 cell count is associated with a higher likelihood of developing neurosyphilis^[5]^).

On the behavioral side, ART influences sexual risk behavior differently depending on the setting,^[10]^ with some evidence pointing toward no change in sexual risk behavior and other suggesting risk compensation.^[11]^ In the Swiss HIV Cohort Study (SHCS), a trend of increasing condomless sex in all transmission groups has been observed. This increase was especially evident in men who have sex with men (MSM): individuals under ART treatment in both stable and casual relationships are using condoms less frequently.^[12]^ Similar patterns of increased condomless sex among MSM have been observed in the United States as well.^[13]^

Finally, ART has a direct effect on HIV coinfections including herpes simplex virus type 2^[14]^ and Hepatitis B Virus.^[15]^ In addition, ART has been shown to have a wide array of targets and functions including activity as antitumor, antibacterial, antifungal, antimalarial, anti-Severe acute respiratory syndrome and anti-influenza agent.^[16]^

Despite several studies examining the interaction between ART and syphilis in HIV-infected individuals, the relationship remains unclear, with associations spanning the entire spectrum, some positive, others negative, and some showing no influence. Several studies suggest that HIV-infected individuals on ART may have better overall treatment response, including a better outcome in neurosyphilis,^[17–19]^ lower rates of syphilis serological failure,^[20]^ shorter time to serological response,^[21]^ and lower adjusted incidence.^[22]^ In contrast, other studies suggest that ART has no influence on syphilis incidence,^[23,24]^ or even increases it,^[25–27]^ while others suggest no effect of ART on treatment or serological failure.^[28,29]^

In light of this complex nature of syphilis coinfection in HIV-positive individuals, we aimed to examine the factors that affect syphilis incidence in the SHCS. We used a large MSM cohort from the SHCS with comprehensive longitudinal data on sexual behavior, treatment regimens and continuation, demographics, treatment response, and immunological profiles. In addition, we assessed the incidence of syphilis in other important transmission groups (heterosexuals [HETs] and intravenous drug users [IDUs]). We aimed to further disentangle the association between ART and syphilis and evaluate whether it is due to immunological factors, behavioral aspects, time trends, or the direct effect of ART.

## 2 Methods

The SHCS is a prospective cohort with ongoing enrollment for HIV-infected individuals in Switzerland since 1988. Clinical, laboratory, and sociodemographics information are collected every 6 months. This includes information about sexual behavior in terms of having a stable or occasional partner(s) in the past 6 months, and if so, whether sex was with or without a condom. All participants provided informed consent, and the study was approved and is conducted per the guidelines of the ethical committees of the respective participating center (refer to http://www.shcs.ch/206-ethic-committee-approval-and-informed-consent for all ethics committee approvals of the participating centers). The SHCS study was shown to be highly representative of the HIV-infected population in Switzerland, including “hard-to-reach” populations.^[30]^

Annual syphilis testing ceased as of 1998 owing to a steady decline in the syphilis infection rate in Switzerland and an internal analysis that revealed that syphilis testing every 2 years imparted no decrement in incidence.^[2]^ Syphilis testing was restarted in 2004, hence only tests taking place thereafter were included here.

Syphilis testing in the SHCS can be divided into 2 categories: nontreponemal and treponemal. The nontreponemal branch contains either Venereal Diseases Research Laboratory or Rapid plasma regains, and the treponemal branch includes Treponema pallidum particle agglutination assay/Treponema pallidum hemagglutination assay, Liaison (CLIA), and Architect (CMIA).

Only individuals with a negative result in a first baseline test according to both methods were included in the analysis. Individuals with missing tests in either arm were excluded, and a case was considered positive only if both markers turned positive.^[31]^ A positive treponemal test with a negative nontreponemal test was considered evidence for previous infections and those individuals/observation time were excluded.

We included in the analysis all available individuals meeting the aforementioned criteria from 2004 to 2014. Owing to the limited number of syphilis cases in HET and IDU, the analysis was later restricted to MSM as they account for the vast majority of cases. This also allowed for a more homogenous population (and consequently a comparable risk behavior). The observation time was defined as the time between the first negative test and either the first positive syphilis test or the last negative test. Finally, only individuals who were observed for a year or more were analyzed. Note that the transmission group of a patient constitutes the most probable route of HIV infection as declared jointly by the patient and the clinician.

## 3 Statistical analysis

Given the inherent interval-censored nature of syphilis incidence (imprecise knowledge of the exact point of time where the infection occurred), we utilized univariable and multivariable parametric interval-censored models with time-fixed and time-varying covariates and an exponential hazard function (as in Ref. ^[15]^). We tested the association of ART (exposure) and syphilis incidence (outcome variable), where ART was coded in several hierarchical ways: ART as a binary variable; ART divided into 4 classes of nucleotide reverse transcriptase inhibitors (NRTI), non-nucleotide reverse transcriptase inhibitors (NNRTI), protease inhibitors (PI), and other HIV antiretroviral drug classes (integrase and fusion inhibitors, primarily Raltegravir [87%]); ART divided into NRTI, PI, other HIV antiretroviral drug classes, and the individual drugs for the NNRTI class (as drugs of this class showed a weak protective association [table S1]). The exposure was subsequently subdivided into ART-treated and suppressed (HIV Ribo-nucleic acid (RNA) ≤ 50 copies/mL) or ART treated and nonsuppressed.

The univariable analysis was constructed to include variables that most likely influence syphilis incidence based on the literature and clinicians’ evaluation. The following covariates were examined in the univariable models: square root transformed CD4, CD8, and nadir CD4 cell count (lowest observed CD4 since enrollment). In addition, baseline CD4 cell count <200 (binary), log transformed HIV RNA copies/mL, smoking (binary), recreational drug use (binary, reflecting no drug use = 0, and any intravenous drug use 1), reporting of condomless sex with a stable or occasional partner (binary), ethnicity (binary, white, and non-white), age at infection (continuous per 5 years), and education (binary, attended a higher education institution).

The inclusion criteria for the multivariable analysis were based on clinical relevance, significance in the univariable analysis (*P* value ≤0.1), and in case of highly correlated variables, only 1 representative was chosen (e.g., for HIV RNA viral load and ART, ART was chosen as it is the explanatory variable of interest). The included variables were immunity markers (CD4 and nadir CD4), behavioral markers (condomless sex with occasional partner, testing rate per year), ART, demographics (age at infection, ethnicity, and last center of follow-up), and calendar year (to account for the time trends of incidence).

For sensitivity analyses, the associations were estimated using a Weibull hazard function and Cox proportional hazard models. Analyses were conducted in Stata 14.1 (Stata Corp., Texas, United States) and R 3.3.1 (R Foundation for Statistical Computing, Vienna, Austria).

## 4 Results

A total of 3575 individuals were routinely tested for syphilis at least once, following a negative baseline test between 2004 and 2014 (median observation start year 2005, inter-quartile range IQR 2004–2007) (Table 1). There were 226 incident syphilis cases reported in 19,041 person-years. The incidence rate was 26.8 cases per 1000 person-years for MSM (95% confidence interval [95% CI] 23.5–30.8), 1.3 (0.49–3.5) for IDU, and 1.8 (1.1–3.0) for HET. This corresponded to 207 (92%) syphilis cases in MSM, 4 (2%) in IDU, and 15 (6%) in HET.

**Table 1:**
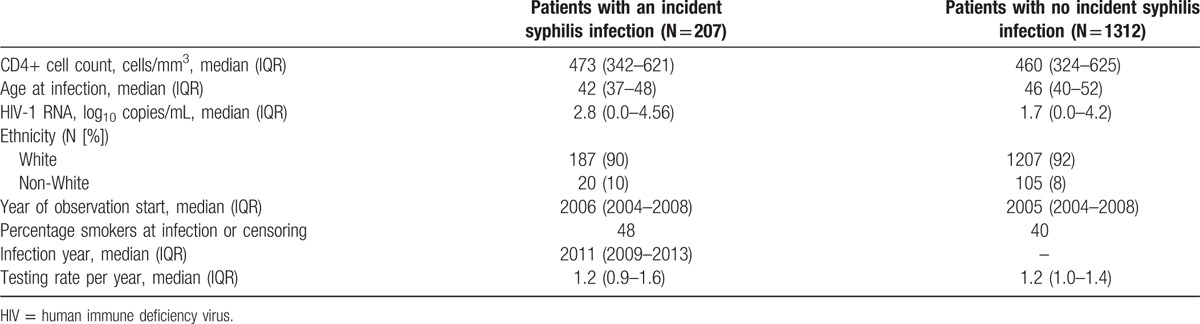
General characteristics of eligible MSM SHCS patients at enrollment.

**Figure 1:**
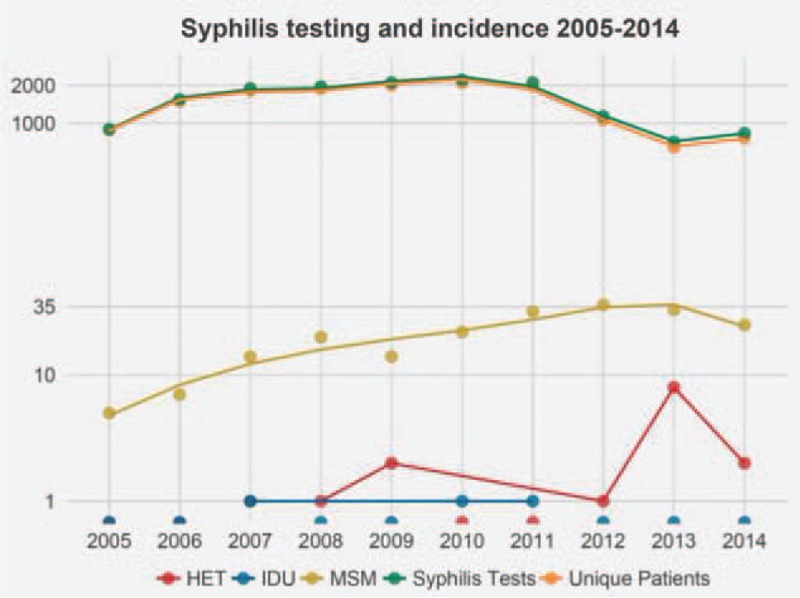
The number of unique Swiss HIV Cohort study patients tested per year and the number of syphilis tests they underwent are shown for the years 2005 to 2014. The number of syphilis cases is shown in 1000 patient-years per transmission group. The vertical axis is log-scaled and dots below 1 represent years with 0 syphilis cases. HET = heterosexual, IDU = intravenous drug use, MSM = men who have sex with men.

**Figure 2:**
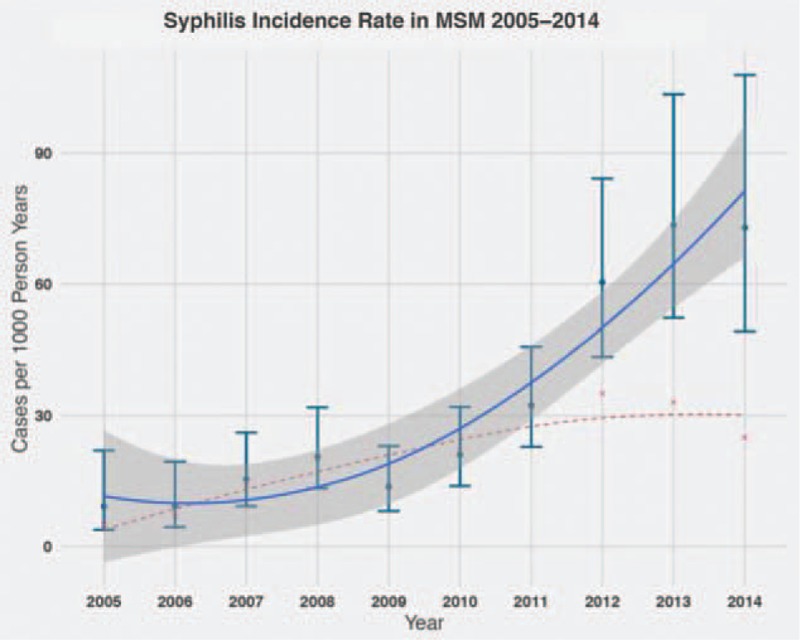
Teal-colored dots and confidence intervals (CIs) represent the incidence rate of syphilis in the Swiss HIV Cohort study between 2005 and 2014, cases per 1000 person-years (for easier trend visualization, the blue line is a natural cubic spline fitted to the incidence rate, the gray shaded region shows the estimated 95% CI). The red dots show the absolute number of incident cases per year (the red line represents a natural cubic spline fitted to the absolute number of cases).

During the study period (2004–2014), we found a significant increase of syphilis incidence over time (Pearson correlation between annual incidence and year 0.9, *P* < 0.01; Figs. 1 and 2, and Tables 2 and 3). We did not observe a clear elevation of syphilis incidence immediately after the year 2008 (the year of the so-called Swiss statement^[32]^). However, the incidence rate of syphilis increased 2-fold by 2012, and a similar trend was reflected in the hazard of acquiring syphilis estimated by the model described in Section 3 (Tables 2 and 3). In addition, we observed that the MSM population is most affected by syphilis and accounts for the majority of incident cases (92%). Finally, the number of cases in IDU has remained constantly low over time, while that of HET seems to be increasing; yet the overall low number of cases does not allow for conclusive assessment, particularly because 2 of the 19 syphilis-infected patients in the IDU and HET transmission group identify as bisexual and another individual of the 19 identifies as homosexual.

**Table 2:**
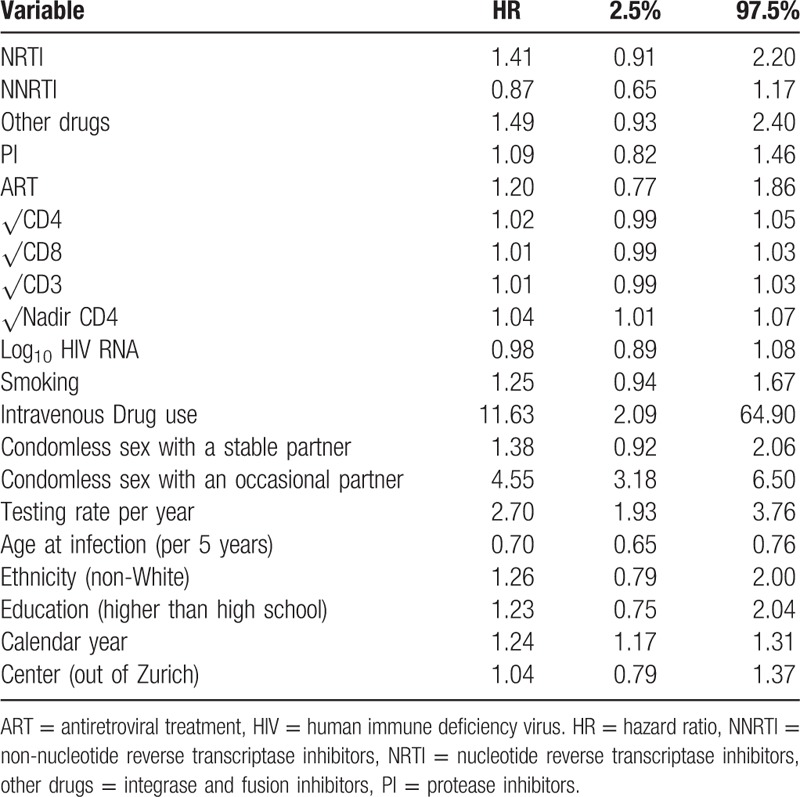
Univariable analysis of factors associated with syphilis incidence in MSM.

**Table 3:**
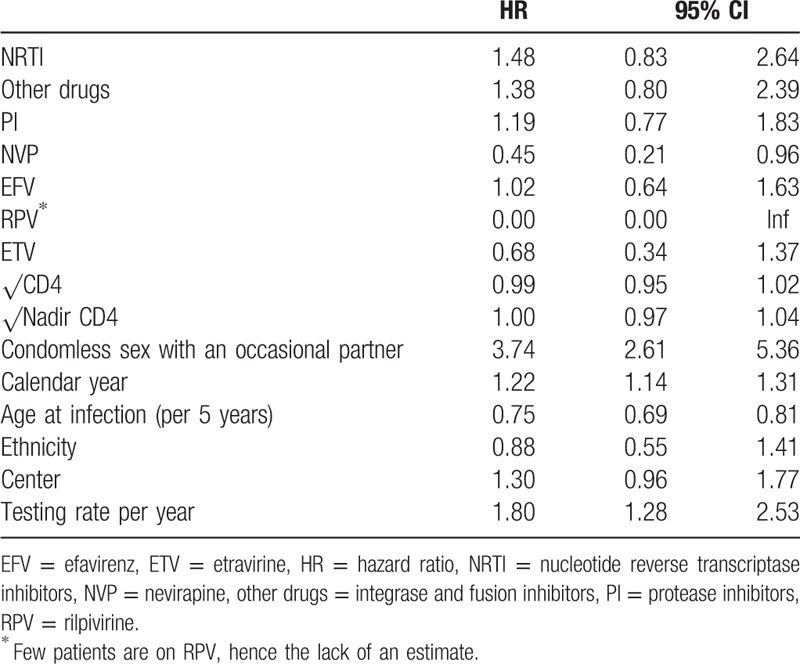
Multivariable analysis of factors associated with syphilis incidence in MSM.

Hereafter, we focus on the MSM population, as it is the group with the highest incidence, and in which public health interventions would have the strongest impact.

We observed no association between being on ART and syphilis incidence (univariable hazard ratio [HR] 1.2, 95% CI 0.8–1.9). Breaking down ART treatment into individual drug classes revealed that NNRTI could potentially have a protective association against syphilis (HR 0.9, 95% CI 0.7–1.2; table S1). Indeed, we found that nevirapine (NVP) was associated with lower syphilis incidence (multivariable HR 0.5, 95% CI 0.2–1.0) (Table 3). We also observed that the protective association of NVP was tightly coupled to viral load suppression (plasma RNA ≤ 50) (NVP and suppressed HR 0.4, 95% CI 0.2–0.9). No protective effect of NVP was present in nonsuppressed patients on NVP (HR 1.0, 95% CI 0.1–7.9). Furthermore, the effect of NVP did not seem to be mediated by immune markers as the association remained even after adjusting for these variables (among others, Tables 2 and 3). Neither Ritonavir-boosted nor nonboosted PI was associated with syphilis incidence.

A decrease in syphilis incidence with increasing age was the only demographic association (univariable HR 0.7, 95% CI 0.7–0.8); neither ethnicity nor education had an effect (Table 3).

On the behavioral side, risk behavior was generally associated with higher incidence of syphilis. A history of condomless sex with an occasional partner increased the hazard of acquiring syphilis (univariable HR 4.6, 95% CI 3.1–6.50). Interestingly, condomless sex with a stable partner was also associated with higher incidence of syphilis (univariable HR 1.4, 95% CI 0.9–2.1); however, this association was not statistically significant. While individuals who reported being in a stable partnership were less likely to engage in condomless sex with occasional partners (odds ratio 0.24, 95% CI 0.24–0.25), 12% reported such behavior. We also observed that intravenous drug use (compared to no drug use) was associated with higher incidence of syphilis (HR 11.6, 95% CI 2.1–65). However, only 9 individuals reported intravenous drug use for 0.07% of the total observation time; hence, this covariate was not included in the multivariable model as a marker of risk behavior. Moreover, smoking was associated with an increased syphilis incidence, yet the association was not statistically significant (HR 1.3, 95% CI 0.9–1.7, Tables 2 and 3). Finally, individuals who tested more frequently for syphilis also had a higher propensity to be infected (univariable HR 2.7, 05% CI 1.9–3.8).

The immunological status represented by square root transformed CD4, CD8, and nadir CD4 cell count was not associated with the hazard of contracting syphilis (Tables 2 and 3).

The estimates were qualitatively similar when using a Cox proportional hazard model or a Weibull hazard function for the current model (results not shown).

## 5 Discussion

In this study, we report on the factors associated with syphilis incidence in the era of highly effective ART in the SHCS. Most prominently, we observed a steady increase of syphilis incidence, with the highest burden falling on MSM. We showed that risk behavior (condomless sex or drug use) and young age were all associated with a higher risk of contracting syphilis, while NVP treatment was associated with a lower risk.

We observed no association between being on ART and syphilis incidence, which implies that immune reconstitution does not provide significant protection against syphilis. The protective effect of NVP was only present in individuals with viral suppression, that is, only NVP that achieved viral suppression (≤50 HIV RNA copies/mL) conferred a protective effect. This suggests the necessity of consistent drug administration and adherence for NVP to exhibit its potential protective effect against syphilis.

Generally, we observed a lower overall incidence rate of syphilis in the SHCS than that of HIV-infected MSM in Canada,^[33]^ the Netherlands,^[34]^ and the United States^[22]^ (27 per 1000 person-years for the SHCS compared to 42, 46, and 62, respectively). We also observed a consistent rise in syphilis incidences in recent years; 25 to 35 per 1000 person-years between 2009 and 2011 compared to 60 to 74 in 2012 to 2014 (Fig. 2). Similar increases have been reported in other countries,^[1,22,34]^ and syphilis incidence remains very high in the MSM community. At the same time, there are more reports of condomless sex in the SHCS^[12]^ and in other countries.^[13]^

Condomless sex remains one of the strongest drivers of syphilis incidence in the SHCS. Consequently, better risk reduction measures are needed. For example, it has been shown that patients who have already had an episode of syphilis are 3 times more likely to be reinfected,^[33]^ implying continued risky behavior tendencies in some populations. Such individuals are prime targets for more frequent screening and other supportive therapy. We observed that individuals who get tested more frequently (i.e., more than once a year during routine screening) are 2 to 3 times more likely to get infected with syphilis. These individuals are probably screened more often due to their self-reported risky sexual behavior (with testing initiated either by themselves or by their clinician), which underlines the importance of frequent testing in high-risk groups. Several studies have demonstrated the benefit of screening for syphilis every 3 months compared to 6 or 12 months.^[35]^ The observation that younger age is associated with higher syphilis incidence should help target intervention efforts.

Our findings are in line with the reported syphilis-incidence trends in non-HIV-infected MSM in Switzerland (BAG Bulletin 2011–2014). This implies that the behavior leading to syphilis infection is not exclusive to HIV-infected individuals. A high syphilis incidence together with the untreated HIV-infected individuals (mostly undiagnosed) could be one of the factors contributing to the still relatively high rate of new HIV infections seen in Switzerland, despite almost fulfilling the World Health Organization 90-90-90 criteria.^[36]^ Yet, the root cause of both syphilis and HIV incidence remains condomless sex. As in our earlier study of condomless sex in the SHCS,^[12]^ it is clear that condomless sex correlates strongly with a general increase of incidence of sexually transmitted infections (STIs). One of the challenges that any STI prevention measure in a high-risk population will face is communicating that the efficacy of HIV treatment as prevention and pre-exposure prophylaxis does not extend beyond HIV, and that one remains vulnerable to other STIs by engaging in condomless sex. A recent meta-analysis showed that MSM receiving pre-exposure prophylaxis were between 11.2 and 44.6 times more likely to get infected with an STI, with syphilis having the strongest likelihood (44.6).^[37]^

One factor that could benefit from further investigation is the study of the circulating syphilis strains of the past years. Multiple reports indicate that there are several circulating *Treponema pallidum* strains with different genetic profiles^[38]^ (e.g., Azithromycin-resistant variants^[39,40]^). This could imply that the increase in syphilis incidence, while readily explainable by an increase in condomless sex, could be further fueled by a shift in the circulating strains of *T. pallidum* that are more transmissible or pathogenic (unfortunately such data are not available in Switzerland).

To our knowledge, there are no known mechanistic effects for NVP against *T. palladium*. Given the observational nature of our study, one cannot exclude the contribution of unobserved confounders to the observed association. Nonetheless, there are reports of other antiretrovirals being effective against both gram-positive and gram-negative bacteria (Shilaih et al submitted). *T. pallidum* remains one of the bacteria that cannot be grown in axenic medium and requires laborious infection of animals (rabbits) to be studied; therefore, it is highly challenging to evaluate the effect of NVP experimentally.

We strived to select a homogenous sample of individuals to minimize demographic and behavioral influence on the associations (hence the focus on MSM); however, this does not rule out the potential presence of other factors not accounted for by the chosen covariates. In other words, we cannot rule out that the observed NVP effect occurred because patients who receive NVP are different from the overall study population in ways not captured by the covariates included in the multivariable analysis. Moreover, while the syphilis testing procedure as described in Section 2 is considered the standard of care worldwide,^[41]^ it is known that false positives as well as false negatives could occur, especially in HIV-infected individuals.^[42,43]^

This study demonstrates that syphilis incidence has been continuously increasing in recent years with the main driver being high-risk behavior. In addition, we demonstrate that ART generally does not provide a protective effect against syphilis. Given these findings, more frequent screening targeted for MSM is needed to limit syphilis spread (and other STIs) and the probably consequent increase of HIV infections. Moreover, we found an unexpected but intriguing protective association of NVP against syphilis incidence, which demands further investigation—epidemiologically and potentially in other settings such as prospective clinical trials. If proven effective, NVP could be recommended to high-risk individuals for its potential to reduce HIV transmission and syphilis acquisition.

## Supplementary Material

Supplemental Digital Content
