# Factors associated with syphilis incidence in the HIV-infected in the era of highly active antiretrovirals: Erratum

**DOI:** 10.1097/MD.0000000000006435

**Published:** 2017-03-10

In the articles “Factors associated with syphilis incidence in the HIV-infected in the era of highly active antiretrovirals”,^[1]^ which appeared in Volume 96, Issue 2 of *Medicine*, Huldrych F. Günthard was listed as a PhD. In fact, the author is an MD.
