# Supplementary material for: Factors associated with syphilis incidence in the HIV-infected in the era of highly active antiretrovirals
Source: Medicine (Baltimore). 2017 Jan 13;96(2):e5849. doi: 10.1097/MD.0000000000005849 (PMC5266180; doi:10.1097/MD.0000000000005849)
Supplement: Supplemental Digital Content [file medi-96-e5849-s001.docx]

Table S1 Multivariable analysis of Factors Associated with Syphilis Incidence in MSM

|  | HR | 95% CI | |
| --- | --- | --- | --- |
| NRTI | 1.56 | 0.89 | 2.73 |
| Other Drugs | 1.30 | 0.78 | 2.16 |
| PI | 1.13 | 0.74 | 1.71 |
| NNRTI | 0.78 | 0.51 | 1.18 |
| √CD4 | 0.99 | 0.95 | 1.02 |
| √Nadir CD4 | 1.00 | 0.97 | 1.04 |
| Condomless sex with an occasional partner | 3.72 | 2.59 | 5.35 |
| Calendar Year | 1.20 | 1.13 | 1.29 |
| Age at infection (per 5 years) | 0.74 | 0.68 | 0.81 |
| Ethnicity | 0.93 | 0.58 | 1.49 |
| Center | 1.30 | 0.96 | 1.77 |
| Testing rate per year | 1.81 | 1.29 | 2.54 |
